# *Dipterocarpus tuberculatus* Roxb. Ethanol Extract Has Anti-Inflammatory and Hepatoprotective Effects In Vitro and In Vivo by Targeting the IRAK1/AP-1 Pathway

**DOI:** 10.3390/molecules26092529

**Published:** 2021-04-26

**Authors:** Haeyeop Kim, Woo Seok Yang, Khin Myo Htwe, Mi-Nam Lee, Young-Dong Kim, Ki Dong Yoon, Byoung-Hee Lee, Sarah Lee, Jae Youl Cho

**Affiliations:** 1 Department of Integrative Biotechnology, Sungkyunkwan University, Suwon 16419, Korea; rlagoduq7283@naver.com (H.K.); real0902@gmail.com (W.S.Y.); 2 Popa Mountain Park, Forest Department, Kyaukpadaung Township, Mandalay Division, Kyaukpadaung 05241, Myanmar; khinmyohtwe007@gmail.com; 3 Department of Hospitality and Culinary, Ansan University, Ansan 15318, Korea; hhong3159@naver.com; 4 Department of Life Science, Hallym University, Chuncheon 200-702, Korea; ydkim@hallym.ac.kr; 5 College of Pharmacy, The Catholic University of Korea, Bucheon 420-743, Korea; kdyoon@catholic.ac.kr; 6 National Institute of Biological Resources, Environmental Research Complex, Incheon 22689, Korea; dpt510@korea.kr

**Keywords:** *Dipterocarpus tuberculatus* Roxb., anti-inflammatory effects, tumor necrosis factor-α, interleukin-1 receptor-associated kinase, activator protein-1 pathway, hepatitis model

## Abstract

*Dipterocarpus tuberculatus* Roxb. has been used traditionally as a remedy for many diseases, especially inflammation. Therefore, we analyzed and explored the mechanism of the anti-inflammatory effect of a *Dipterocarpus tuberculatus* Roxb. ethanol extract (Dt-EE). Dt-EE clearly and dose-dependently inhibited the expression of pro-inflammatory cytokines such as IL-6, TNF-α, and IL-1β in lipopolysaccharide (LPS)-treated RAW264.7 cells. Also, Dt-EE suppressed the activation of the MyD88/TRIF-mediated AP-1 pathway and the AP-1 pathway related proteins JNK2, MKK4/7, and TAK1, which occurred as a result of inhibiting the kinase activity of IRAK1 and IRAK4, the most upstream factors of the AP-1 pathway. Finally, Dt-EE displayed hepatoprotective activity in a mouse model of hepatitis induced with LPS/D-galactosamine (D-GalN) through decreasing the serum levels of alanine aminotransferase and suppressing the activation of JNK and IRAK1. Therefore, our results strongly suggest that Dt-EE could be a candidate anti-inflammatory herbal medicine with IRAK1/AP-1 inhibitory and hepatoprotective properties.

## 1. Introduction

Inflammation is a response of the human immune system that occurs when immune cells fight with pathogens invading from the outside [1]. The immune system is necessary to defend human bodies from pathogens and viruses and maintain healthy lives. When an inflammatory reaction occurs, five symptoms appear that include fever, pain, redness, swelling, and loss of function. These reactions are obviously beneficial, but when they are not properly controlled or become dysregulated, acute inflammation can develop into chronic inflammation [2,3], which can be harmful and cause many diseases, including myocardial infarction, atherosclerosis, autoimmune diseases, and cancers [4,5].

The interaction between innate immune receptors called pattern recognition receptors (PRRs), and conserved structures called pathogen-associated molecular patterns (PAMPs) induces the non-specific responses of innate immunity [6]. Various ligands and receptors, such as lipopolysaccharide (LPS), the main constituent of the outer membrane of Gram-negative bacteria, and toll-like-receptor 4 (TLR4), activate cells and induce inflammatory responses [7,8]. TLRs were the first PRRs to be identified and play a key role in pathogen recognition [9]. They dimerize and interact with each specific ligand. This binding between receptor and ligand activates several intracellular signals that stimulate inflammation. Among several types of PRRs and PAMPs, the response to TLR4 and LPS in macrophages is the most effective in inducing inflammation. TLR4 binding to LPS induces conformational changes in the intracellular domain and recruits scaffolding proteins such as toll/interleukin-1 receptor-domain-containing adapter-inducing interferon-β (TRIF) and myeloid differentiation primary response 88 (MyD88) [10,11,12]. Such LPS/TLR4-induced processes trigger multiple signaling pathways, including the activator protein-1 (AP-1), cAMP response element-binding protein (CREB), and nuclear factor kappa B (NF-κB) pathways [13]. The TRIF/MyD88-dependent pathway recruits the interleukin-1 receptor-associated kinase 1/4 (IRAK1/4) and TGF-β activated kinase 1 (TAK1), and TAK1 then activates the mitogen-activated protein kinase (MAPK) pathway and the c-Jun N-terminal kinase (JNK) [14,15]. Finally, c-Jun, a protein that acts as an early response transcription factor for AP-1, enters the nucleus as the result of this activation cascade, which leads to AP-1-induced gene expression. The AP-1-related pro-inflammatory cytokines include TNF-α, IL-6, and IL-1β, and upon AP-1 activation, their expression increases [16,17]. These pro-inflammatory cytokines are mainly produced by activated macrophages, and they promote inflammatory responses [18].

*Dipterocarpus tuberculatus* is a genus of flowering plants and the type genus of the family Dipterocarpaceae. As a representative medicinal herb [19], it is prescribed for various diseases in Southeast Asia, especially Cambodia, Thailand, Laos, and Vietnam [20]. This plant has been used for leishmanicidal, anti-septic and anti-inflammatory purposes [21] and is also traditionally prescribed for curing skin inflammation, bronchial infections, colitis, and anxiety [22]. Many quinone analogs have been purified from the heartwood of this plant and these compounds are considered to function as antioxidative components [23]. Particularly, this plant has been reported to reduce NF-κB-mediated inflammatory responses in vitro and in vivo [24]. We have continued to study the ethnopharmacological roles of Dt-EE and found that Dt-EE also exerts an anti-inflammatory effect by inhibiting the AP-1 pathway. In addition, we confirmed that Dt-EE has anti-inflammatory effects in both gastritis and LPS/D-galactosamine (D-GalN)-triggered hepatitis.

In the in vitro and in vivo experiments reported here, we discovered the mechanism of action and direct pharmacological target enzymes involved in anti-inflammatory and hepatoprotective responses of Dt-EE.

## 2. Results

### 2.1. Effects of Dt-EE on Pro-Inflammatory Cytokines at a Trascriptional Level

**Figure 1:**
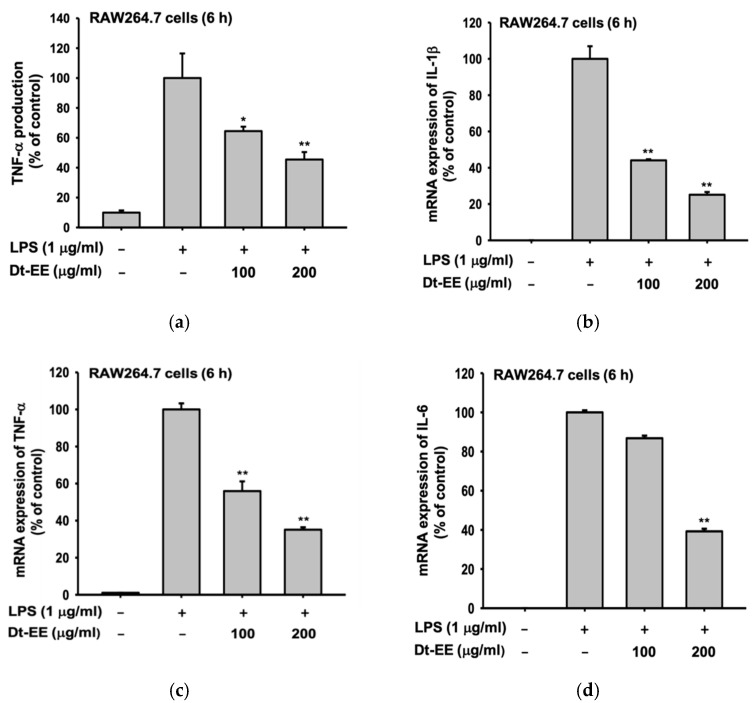
Anti-inflammatory effects of Dt-EE on pro-inflammatory cytokines. (**a**) The production of TNF-α in LPS-induced RAW264.7 cells with and without Dt-EE for 6 h was detected by ELISA. (**b**–**d**) The mRNA expression levels of pro-inflammatory cytokines, IL-1β, TNF-α and IL-6, were measured using quantitative real-time PCR. *: *p* < 0.05 and **: *p* < 0.01 compared to the induced group.

It has been already reported that Dt-EE has no cytotoxicity in RAW264.7 cells or HEK293 cells [24]. Therefore, in this experiment, we used ELISA to determine the expression rate of TNF-α as a way to confirm the anti-inflammatory effect of Dt-EE. As Figure 1a shows, LPS treatment induced a high expression level of TNF-α and Dt-EE reduced that increased level by 40% or more in a dose-dependent manner (Figure 1a). In addition, we used quantitative real-time PCR after extracting mRNA from the cells and synthesizing cDNA. Our results confirm that the mRNA expression levels of pro-inflammatory cytokines (IL-1β, TNF-α, and IL-6) increased rapidly following LPS treatment and then decreased following treatment with Dt-EE (Figure 1b–d).

### 2.2. Effects of Dt-EE on the Activation of the AP-1 and CREB Pathways

**Figure 2:**
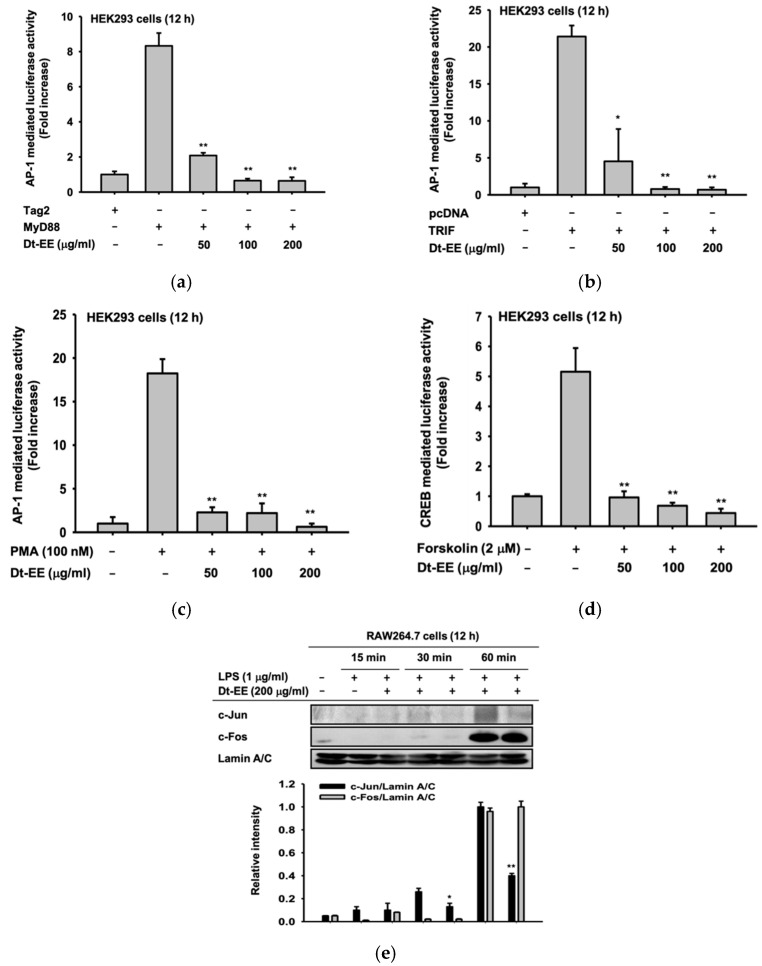
Effect of Dt-EE on inflammatory transcriptional activation. (**a,b**) FLAG-MyD88 or CFP-TRIF was transfected in HEK293 cells using plasmid constructs of AP-1-Luc and β-gal (as a transfection control) for 24 h, followed by treatment with Dt-EE (0–200 µg/mL) for an additional 24 h. (c) HEK293 cells were transfected with plasmid constructs of AP-1-Luc and β-gal (as a transfection control) for 24 h, followed by treatment with Dt-EE (0–200 µg/mL) and PMA (100 nM) for an additional 24 h. (**d**) HEK293 cells were transfected with CREB-Luc and β-gal and incubated for 24 h. Then, forskolin (2 µM) and Dt-EE (0–200 µg/mL) were administered according to the conditions shown. Luciferase activity was measured using a luminometer and normalized to that of β-gal. (**e**) The nuclear translocation levels of c-Jun and c-Fos, subunits of AP-1, and Lamin A/C were determined by western blotting of nuclear fractions from RAW264.7 cells with and without LPS (1 µg/mL) and Dt-EE (0–200 µg/mL) treatment for the indicated times. *: *p* < 0.05 and **: *p* < 0.01 compared to the induced group.

Next, we worked to determine which inflammatory pathways were inhibited by the treatment of Dt-EE. To do that, we overexpressed the MyD88 and TRIF adaptor molecules to induce activation of the AP-1 pathway. We also treated phorbol 12-myristate 13-acetate (PMA, 100 nM) to activate the MAPK/ERK pathway and forskolin to induce the CREB pathway [25]. The luciferase reporter gene assay results strongly indicated that the activities of AP-1 and CREB transcription factors can be reduced by Dt-EE treatment (Figure 2a–d). In that way, we verified that Dt-EE inhibits the activity of the AP-1 and CREB pathways. To confirm those discoveries at the molecular level, we examined the nuclear translocation of two subunits of the AP-1 transcription factor, c-Jun and c-Fos. When the AP-1 pathway is activated, subunits of AP-1 translocate into the nucleus, and then transcription proceeds accordingly. However, in cells treated with Dt-EE, the amount of c-Jun expression in the nucleus was significantly reduced (Figure 2e). Thus, we found that Dt-EE inhibits the inflammatory response by inhibiting the activity of the AP-1 pathway, which is one of the inflammation-inducing pathways.

### 2.3. Effects of Dt-EE on the Enzyme Activity of IRAK1 and Regulation of the AP-1 Pathway

To explore more deeply, we conducted an experiment to discern which of several proteins involved in the AP-1 pathway Dt-EE inhibits. Using western blotting analyses, we examined cells treated with LPS with and without Dt-EE and observed the total and phosphorylated proteins involved in the AP-1 pathway over time. Because the degree of nuclear translocation of c-Jun, a transcription factor, decreased upon treatment with Dt-EE, we examined the active forms of upstream factors in the c-Jun-related pathway: JNK1/2, MKK4/7, TAK1, and IRAK1 [26]. The amount of phosphorylated JNK2 was decreased at the 30 and 60 mi time points, and the amount of phosphorylated MKK4/7 was decreased at 5, 15, 30, and 60 min. The amount of phosphorylated TAK1 was decreased at 30 min. We also found that the total expression level of IRAK1, the most upstream factor of these proteins, was higher 5 min after Dt-EE treatment than it was in the non-treated group.

**Figure 3:**
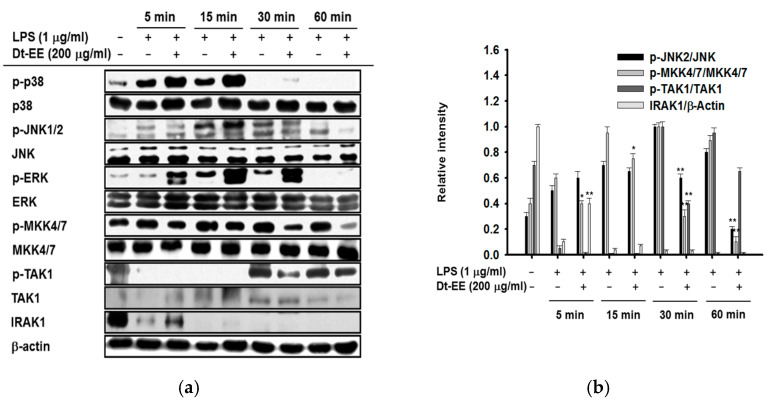

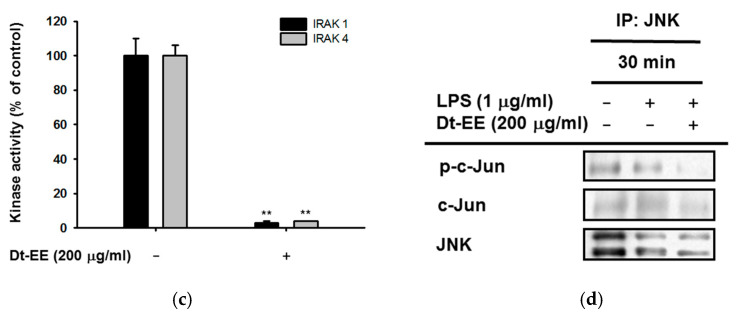
Effect of Dt-EE in activating the AP-1 pathway. (**a**) The total and phosphorylated proteins of the AP-1 pathway (p38, JNK1/2, ERK, MKK4/7, TAK1, and IRAK1) and β-actin were detected by western blotting analyses. (**b**)The band intensities were calculated using ImageJ. (**c**) The kinase activities of IRAK1 and IRAK4 were measured by a kinase profiler service from Millipore. (**d**) The binding levels of JNK and c-Jun were detected in whole cell lysates by immunoprecipitation with anti-JNK and immunoblotting with antibodies to p-c-Jun, c-Jun, and JNK. *: *p* < 0.05 and **: *p* < 0.01 compared to the induced group.

Usually, IRAK1 and IRAK4 bind with MyD88, but when a cell is activated, IRAK4 activates IRAK1, and IRAK1 undergoes autophosphorylation and kinase activity and is then degraded [27]. In terms of this, the increased level of total IRAK1 by Dt-EE strongly indicates that Dt-EE can suppress kinase activity and autophosphorylation of IRAK1, leading to its upregulation (Figure 3a, Left panel). We also confirmed significant differences between the proteins by comparing the amount of expression, as measured by the band intensity (Figure 3b, Right panel). Therefore, we can confirm that the kinase activity of IRAK1 and IRAK4 was decreased by Dt-EE (Figure 3b). Furthermore, the expression of phosphorylated c-Jun in LPS-induced cells decreased when Dt-EE was administered, compared with the non-treated group. The ability of immunoprecipitated JNK and c-Jun to form a molecular complex is suppressed by Dt-EE, which implies that the inhibition of direct kinase activity could affect the formation of inflammatory signaling complexes and the phosphorylation of downstream substrates (Figure 3c). In conclusion, we found that Dt-EE inhibits the kinase activity of IRAK1 and IRAK4, thereby inhibiting the activation of TAK1, MKK4/7, and JNK2.

### 2.4. Effect of Dt-EE in an In Vivo Hepatitis Model in Mice

**Figure 4:**
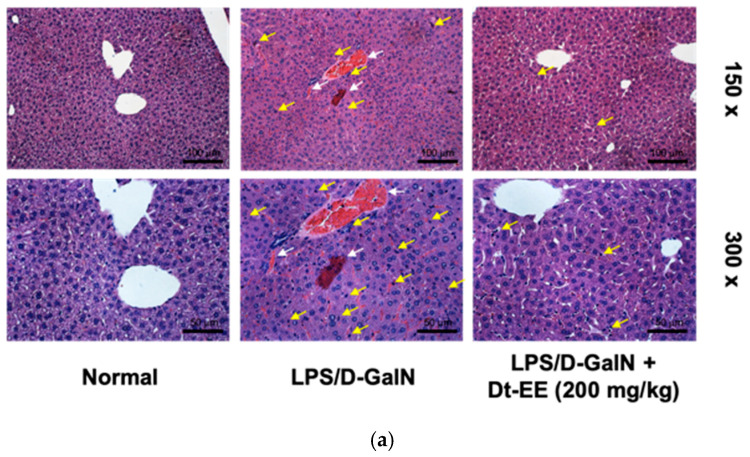

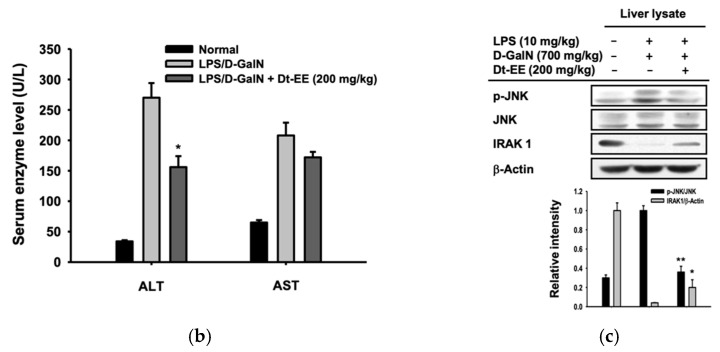
Effect of Dt-EE in a mouse model of LPS/D-GalN-induced hepatitis. (**a**) Histological analysis was conducted by H&E staining experiment with the liver tissues of LPS/D-GalN-induced mice. Mice livers were excised and stained with hematoxylin and eosin (150× and 300×). White arrows indicate bleeding and yellow arrows indicate neutrophilic recruitment. (150×–Scale bar: 100 µm, 300×–Scale bar: 50 µm). (**b**) The expression levels of serum enzymes, ALT and AST, were measured using a Roche Modular spectrophotometric autoanalyzer. (**c**) The total and phospho-protein levels of JNK and IRAK1, and β-actin were determined by an immunoblotting analysis using liver lysate from mice with induced hepatitis with and without Dt-EE treatment (200 mg/kg). *: *p* < 0.05 and **: *p* < 0.01 compared to the induced group.

So far, we have confirmed at the molecular level that Dt-EE has anti-inflammatory effects in vitro. To determine whether Dt-EE has anti-inflammatory effects in vivo, we examined whether orally administered Dt-EE for six days can reduce liver damage triggered by LPS-D/GalN. Acute liver injuries are primarily related to LPS, a well-known gram-negative bacterial membrane component, that often plays an important role in the initiation of endotoxic damage and induces the activity of inflammatory cytokines, which cause liver tissue damage [28]. D-Galactosamine, a liver-specific toxin, increases the ability of endotoxins such as LPS to induce liver toxicity within a few hours and selectively depletes free nucleotides made by hepatocytes to inhibit the synthesis of mRNA and protein [29]. Therefore, the LPS/D-GalN-induced hepatitis mouse model is widely used to induce the necrosis of hepatocytes [30]. Figure 4a illustrates the results of H&E staining of livers from the hepatitis model mice at 150× and 300× magnification. When the livers were damaged with LPS, the numbers of bleeding, the death of hepatocytes, and immune cell infiltration (e.g., neutrophilic recruitment) were clearly more remarkable than those in the normal group (Figure 4). In fact, H&E staining display clear difference between normal and the LPS/D-GalN groups, while the lesion of acute liver injury was suppressed in mice orally administered with Dt-EE (200 mg/kg) (Figure 4a).

Next, we checked the serum expression levels of alanine aminotransferase (ALT) and aspartate aminotransferase (AST). These two enzymes originally exist in the cytoplasm of cells, but if liver cells are damaged, they are released into the blood [31]. Therefore, we determined the degree of liver damage by measuring the concentration of those two enzymes in the blood. The serum of mice fed Dt-EE showed a significant decrease in ALT (Figure 4b).

As the last experiment, livers of hepatitis mouse model were ground, and the amount of protein expression was measured using liver lysate. As confirmed above (see Figure 3a), the level of the phosphorylated form of JNK was confirmed in vivo, and the amount of phosphorylated JNK in the liver lysate tended to decrease in the mice that received Dt-EE before the induction of hepatic injury. Also, the total IRAK1 expression levels in the liver lysates increased in mice that received Dt-EE (Figure 4c).

## 3. Discussion

The demand for natural plant extracts is increasing because they are deemed safe and have few side effects when they are used as pharmaceuticals [32,33,34]. Accordingly, studies on plant extracts that have anti-inflammatory, antioxidant, and antibacterial effects on various cells are being conducted continuously. Among those plant extracts, we chose *Dipterocarpus tuberculatus*, which has traditionally been used medicinally in various countries of Southeast Asia, for anti-inflammatory experiments. We found evidence of anti-inflammatory efficacy by identifying the inflammatory pathway and direct target protein that this extract inhibits [18].

Dt-EE reduced the expression of IL-1β, TNF-α, and IL-6 at the mRNA level in RAW264.7 cells induced by LPS (Figure 1a–d). These pro-inflammatory cytokines can affect the process of pathological pain and promote inflammation. In the case of TNF-α, the amount of expression was repeatedly confirmed using ELISA, and all three cytokines were confirmed to be reduced by Dt-EE. TNF-α is a one of the cytokines that induces the acute phase of inflammation that is accompanied by fever [35]. To confirm which inflammatory pathway was inhibited, we performed a luciferase assay and confirmed that Dt-EE diminished PMA-induced and MyD88- or TRIF-mediated AP-1 activation and the forskolin-induced CREB pathway (Figure 2a–d). We continued our study by focusing on a well-known inflammation inducing signal in the AP-1 pathway. We checked the nuclear translocation of c-Jun and c-Fos, subunits of the AP-1 transcription factor and found that the degree of translocation of c-Jun was significantly decreased following treatment with Dt-EE (Figure 2e).

As we previously described, we checked the total and phosphorylated proteins involved in the AP-1 pathway to confirm how Dt-EE suppressed the pathway and determine which protein is the direct target protein of Dt-EE. We found that Dt-EE suppressed the activation of IRAK1, TAK1, MKK4/7, and JNK2 and the kinase activities of IRAK1 and IRAK4 (Figure 3a,b). Also, Dt-EE inhibited the binding of immunoprecipitated JNK and c-Jun (Figure 3c). IRAK families, such as IRAK1 and IRAK4, form a complex with TRAF6-TAK1-TAB2-TAB3 on the membrane. To initiate inflammation, it is important for IRAK1 to be phosphorylated on threonine 209 by IRAK4 and then ubiquitinated and degraded. Therefore, degradation of IRAK1 is necessary to activate the processes downstream of IRAK1, such as the phosphorylation of TAK1 and the translocation of IRAK complexes from the membrane to the cytosol [36].

In our previous paper, we confirmed in vivo that Dt-EE inhibits gastritis. In this study, we confirmed the hepatoprotective activity of Dt-EE in LPS/D-GalN-treated mice. The liver plays an important role in nutrient metabolism, detoxification, and the regulation of circulation. Acute hepatitis causes a sudden deterioration of liver function in patients without a history of liver diseases, and it is known to have high mortality and poor prognosis [37]. Furthermore, if acute hepatitis persists and develops into chronic hepatitis, it carries an increased risk leading to developing liver cancer. We used H&E staining, ALT enzyme levels in serum, and western blotting analyses to confirm our results (Figure 4a–c). High serum levels of ALT and AST correlate with a high likelihood of liver disease and liver-related mortality [38]. In conclusion, we here verified that Dt-EE has hepatoprotective effects.

**Figure 5:**
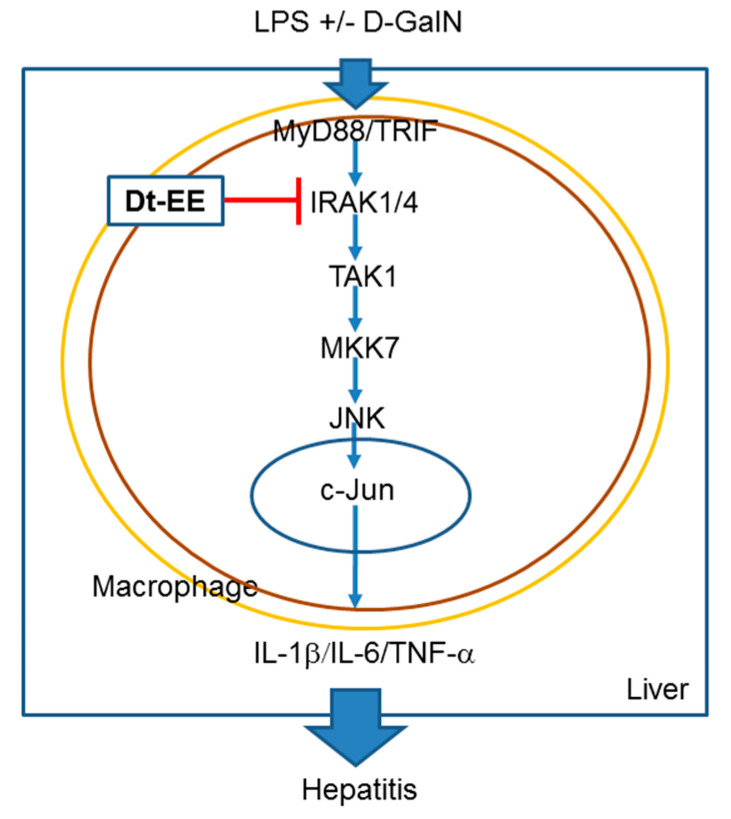
Scheme for Dt-EE-mediated anti-inflammatory activity along the AP-1 signaling pathway.

In conclusion, we have demonstrated that Dt-EE suppresses the kinase enzyme activity of IRAK1, which leads to the decreased activation of the AP-1-mediated inflammatory response, as summarized in Figure 5. Finally, this paper confirmed that Dt-EE is able to show reduction of AP-mediated inflammatory responses under LPS stimulation condition and to exert hepatoprotective activity against acute liver injury triggered by LPS/D-GalN treatment.

## 4. Materials and Methods

### 4.1. Materials and Reagents

A 95% ethanol extract was made using leaves and twigs of *Dipterocarpus tuberculatus* obtained from the Popa Mountain National Park (Mandalay Prov., Myanmar), in August 2011, as reported previously [24]. This plant was identified by Prof. Yong Dong Kim (Hallym University, Chuncheon, Korea). A voucher specimen (number: Cho S.H. et al. MM208) was deposited in the herbariums of Hallym University and National Institute of Biological Resource (Incheon, Korea). Phytochemical fingerprint has been reported previously [24]. LPS (*E.coli* 0111:B4), polyethyleneimine (PEI), phorbol 12-myristate 13-acetate (PMA), forskolin, and D-(+)-galactosamine hydrochloride were purchased from Sigma Chemical Co. (St. Louis, MO, USA). RAW264.7 cells (ATCC number TIB-71) and HEK293 cells (ATCC number CRL-1573) were purchased from the American Type Culture Collection (ATCC) (Rockville, MD, USA). Fetal bovine serum (FBS), Roswell Park Memorial Institute (RPMI) 1640 medium, Dulbecco’s Modified Eagle’s medium (DMEM), phosphate buffered saline (PBS), and TRIzol reagent were purchased from Gibco (Grand Island, NY, USA). c-Jun, c-Fos, Lamin A/C, β-actin, IRAK1, IRAK4, p38, JNK1/2, ERK, MKK4/7, TAK1, and their phospho-specific antibodies were purchased from Cell Signaling Technology (Beverly, MA, USA) or Santa Cruz Biotechnology Inc. (Santa Cruz, CA, USA). A TNF-α ELISA kit (Quantikine^TM^ ELISA, Cat No.: MTA00B) was purchased from R&D Systems (Minneapolis, MN, USA).

### 4.2. Cell Culture

RAW264.7 cells were cultured in RPMI 1640 medium supplemented with 10% inactivated FBS, glutamine, and 1% antibiotics (streptomycin and penicillin). HEK293 cells were cultured in DMEM supplemented with 5% inactivated FBS, glutamine, and 1% antibiotics (streptomycin and penicillin). Both cells were incubated at 37 °C under 5% CO_2_.

### 4.3. Animals

Male C57BL/6 mice (6–8 weeks old, 17–20 g) were purchased from OrientBio (Sungnam, Korea) and housed in groups of 5 mice under standard conditions. Water and feed from Samyang (Daejeon, Korea) were given *ad libitum*. Animals for experiments were bred in accordance with the guidelines specified by the National Institute of Health for the Care and Use of Laboratory Animals (NIH Publication 80-23, revised in 1996), and all in vivo experiments were conducted according to guidelines established by the Institutional Animal Care and Use Committee at Sungkyunkwan University (Suwon, Korea; approval ID: SKKUIACUC2020-11-08-1).

### 4.4. Enzyme-Linked Immunosorbent Assay (ELISA)

For this assay, 100 µL of RAW264.7 cells were seeded at a concentration of 1 × 10^6^ cells/mL per well in a 96-well plate. Dt-EE (0–200 µg/mL) was administered at a concentration four times higher than the indicated concentration, and after 30 min, LPS was also added at four times the target concentration of 1 µg/mL. 100 µL of the supernatants were transferred to an ELISA plate, and TNF-α production levels were determined using an ELISA kit according to the manufacturer’s instructions.

### 4.5. mRNA Analysis Using Quantitative Real-time Polymerase Chain Reaction (PCR)

After RAW264.7 cells were plated in 12-well plates at 1 × 10^6^ cells/mL, Dt-EE (0–200 µg/mL) was administered, followed by treatment with 1 µg/mL of LPS. After incubation for 6 h, total mRNA was extracted using TRIzol reagent, and cDNA was synthesized as previously reported [39,40]. The quantifications of TNF-α, IL-1β, and IL-6 were obtained using quantitative real-time PCR with SYBR Premix Ex Taq (Takara, Shiga, Japan) on a real-time thermal cycler (Bio-Rad, Berkeley, CA, USA) and following the manufacturer’s instruments. The primers used in this experiment are listed in Table 1.

### 4.6. Plasmid Transfection and Luciferase Reporter Gene Activity Assay

HEK293 cells were plated in 24-well plates at 2 × 10^5^ cells/mL and incubated overnight. Then, the AP-1-Luc gene or CREB-Luc gene, MyD88 or TRIF, and genes encoding β-galactosidase were transfected at a ratio of 2:2:1 in 0.8 µg of total genes using the polyethyleneimine method [41]. After incubation at 37 °C under 5% CO_2_ for 24 h, the transfected cells were treated with Dt-EE (50–200 µg/mL), PMA (100 nM), or forskolin (2 µM) and incubated for 6 h. Then the medium was suctioned completely, and 300 µL of luciferase cell lysis buffer (10 mM KH_2_PO_4_, 1mM EDTA in H_2_O) was added to each well of the plate, which was then frozen at −70 °C for 3 h or more. The frozen plate was allowed to thaw at room temperature, which completely lysed the cells. The indicated amounts of lysed cells were plated in a white 96-well plate and reacted 1:1 with luciferin or β-galactosidase. Luminescence was measured using a Synergy HT Multi-Mode Microplate Reader (BioTek Instruments, Inc., Winooski, VT, USA).

### 4.7. Nuclear Fraction, Western Blotting Analysis, and Immunoprecipitation

Nuclear lysates were prepared using the following steps. RAW264.7 cells (2.5 × 10^6^ cells/mL) were plated in 6-cm plates and incubated overnight at 37 °C under 5% CO_2_. Dt-EE (200 µg/mL) was administered 30 min before LPS treatment. LPS (1 µg/mL) was administered and incubated for the designated time: 15 min, 30 min, or 60 min. The cells were washed with cold PBS, scraped into a 1.5 mL tube, centrifuged at 8000 rpm for 5 min at 4 °C, and suctioned. Then, 150 µL of homogenization buffer A (1 M Tris-HCl pH 8.0, 0.2 M EGTA, 0.2 M EDTA, 0.1 M DTT, 0.1 M PMSF, 2 µg/mL aprotinin, 2 µg/mL leupeptin) were added to the cells, and the cells were sonicated for 10 s at 10% power. The cells were centrifuged at 8000 rpm for 15 min at 4 °C and suctioned. Then, 50 µL of homogenization buffer B (1 M Tris-HCl pH 8.0, 0.2 M EGTA, 0.2 M EDTA, 0.1 M DTT, 0.1 M PMSF, 2 µg/mL aprotinin, 2 µg/mL leupeptin, 100% Triton X-100) were added to the cells and suspended. The protein concentration was calculated using the bovine serum albumin standard curve with homogenization buffer B (0–20 µg/mL). Sampling was performed using cell lysates obtained by this method, and the samples were analyzed using western blotting, as previously described [42,43].

RAW264.7 cells (2.5 × 10^6^ cells/mL) were plated in 3-cm plates and incubated overnight at 37 °C under 5% CO_2_. Dt-EE (200 µg/mL) was administered, and after 30 min, LPS (1 µg/mL) was administered and processed for the designated time. Whole cell lysates and liver lysates from the hepatitis model mice were prepared for western blotting, and they were analyzed using specific antibodies diluted in 3% BSA buffer (1:2500) for each target protein, as previously described [42].

For the immunoprecipitation analysis, equal amounts of protein (1000 ng) from RAW264.7 cells lysates treated with LPS (1 µg/mL) for 30 min and untreated controls were prepared. Pre-cleared samples were incubated with 0.4 µL of antibodies to IgG or 3 µL of antibodies to JNK overnight at 4 °C. Complexes of proteins and antibodies were mixed with 50 µL of protein A-coupled Sepharose beads (50% *v*/*v*) and rotated for 4 h at 4 °C. Thereafter, they were washed 5 times with a buffer (50 mM Tris-HCl pH 7.5, 20 mM NaF, 25 mM β-glycerol phosphate pH 7.5, 120 mM NaCl, 2% NP-40, phosphatase/protease inhibitors), and 70 µL of 2× sample buffer (25% glycerol, 2% SDS, 60 mM Tris-HCl pH 6.8, 5% 2-mercaptoethanol, 0.1% bromophenol blue) was added. The samples were heated at 90 °C for 3 min, and the supernatants were then examined by western blotting.

### 4.8. IRAK1 and IRAK4 Kinase Assay

To measure the ability of Dt-EE to inhibit the activity of the IRAK1 and IRAK4 enzymes, we used a kinase profiler service from Millipore (St. St. Louis, MO, USA). IRAK1 and IRAK4 (human) (1–5 mU) were incubated with a reaction buffer for a final reaction volume of 25 µL, and the reaction was started by adding MgATP. The reacted samples were incubated at room temperature for 40 min, and then the reaction was completed by adding 5 mL of 3% phosphoric acid solution. 10 µL of the reaction products were then spotted onto a P30 filtermat and washed three times for 5 min in 75 mM phosphoric acid and once in methanol before drying and scintillation counting.

### 4.9. LPS/D-Galactosamine-Induced Hepatitis Mouse Model

Mice (5 per group) were injected orally with Dt-EE (200 mg/kg) once a day for 6 days using feeding needles. One hour after the last oral injection of Dt-EE, LPS (10 mg/kg) and D-GalN (700 mg/kg) were injected intraperitoneally to induce hepatitis. One hour later, the mice were sacrificed, and experiments were conducted. Blood was obtained by cardiac puncture, and serum was collected by centrifuging the blood at 3000 rpm for 15 min. The livers were excised, rinsed with PBS, and kept at −70 °C. The ALT and AST levels were used as hepatotoxicity indicators and measured quantitatively by following the International Federation of Clinical Chemistry standard method on a Roche Modular spectrophotometric autoanalyzer (Roche, Welwyn Garden, UK).

### 4.10. Histopathology

The mouse livers were excised and fixed with 10% formalin for 1 week. They were then cut into 4 µm sections, placed in embedding cassettes, and embedded with paraffin. After staining with hematoxylin and eosin, the tissues were placed between glasses and observed at 150 and 300 magnification using a microscope.

### 4.11. Statistical Analysis

Data are expressed as the means ± standard error (SEM), as calculated from at least three independent experiments, each performed with n = 3 or n = 5, or from representative data from three different experiments with similar results. For statistical comparisons, the results were analyzed using Kruskal-Wallis/Mann-Whitney testing. A *p* < 0.05 was considered statistically significant. All statistical tests were conducted in SPSS (SPSS Inc., Chicago, IL, USA).

## Figures and Tables

**Table 1:** Primers sequences for the analysis of mRNA prepared from RAW264.7 cells used in quantitative real-time PCR.

Gene	Direction	Sequences (5′ to 3′)
IL-1β	Forward Reverse	GTGAAATGCCACCTTTTGACAGTG CCTGCCTGAAGCTCTTGTTG
TNF-α	Forward Reverse	TGCCTATGTCTCAGCCTCTT GAGGCCATTTGGGAACTTCT
IL-6	Forward Reverse	GACAAAGCCAGAGTCCTTCAGAGA CTAGGTTTGCCGAGTAGATCTC
GAPDH	Forward Reverse	CACTCACGGCAAATTCAACGGCAC GACTCCACGACATACTCAGCAC

## Data Availability

The data used to support the findings of this study are available from the corresponding author upon request.

## Sample Availability

Sample of the compound, Dt-EE, is available from the authors.
